# Auranofin repurposing for lung and pancreatic cancer: low CA12 expression as a marker of sensitivity in patient-derived organoids, with potentiated efficacy by AKT inhibition

**DOI:** 10.1186/s13046-024-03012-z

**Published:** 2024-03-22

**Authors:** Christophe Deben, Laurie Freire Boullosa, Felicia Rodrigues Fortes, Edgar Cardenas De La Hoz, Maxim Le Compte, Sofie Seghers, Marc Peeters, Steve Vanlanduit, Abraham Lin, Krijn K. Dijkstra, Paul Van Schil, Jeroen M. H. Hendriks, Hans Prenen, Geert Roeyen, Filip Lardon, Evelien Smits

**Affiliations:** 1 Center for Oncological Research (CORE), Integrated Personalized & Precision Oncology Network (IPPON), University of Antwerp, Wilrijk, Belgium; 2 Industrial Vision Lab, University of Antwerp, Wilrijk, Belgium; 3 Plasma Lab for Applications in Sustainability and Medicine ANTwerp (PLASMANT), University of Antwerp, Wilrijk, Belgium; 4 Department of Molecular Oncology and Immunology, the Netherlands Cancer Institute – Antoni van Leeuwenhoek Hospital, Amsterdam, The Netherlands; 5 Oncode Institute, Utrecht, The Netherlands; 6 Department of Thoracic and Vascular Surgery, Antwerp University Hospital, Edegem, Belgium; 7 Department of Oncology, Multidisciplinary Oncological Center Antwerp, Antwerp University Hospital, Antwerp, Belgium; 8 Department of Hepatobiliary Transplantation and Endocrine Surgery, University Hospital Antwerp (UZA), Edegem, Belgium; 9 Center for Cell Therapy and Regenerative Medicine, Antwerp University Hospital, Edegem, Belgium

**Keywords:** Auranofin Repurposing, NSCLC and PDAC Therapy, RNAseq Biomarkers, Drug Synergy

## Abstract

**Background:**

This study explores the repurposing of Auranofin (AF), an anti-rheumatic drug, for treating non-small cell lung cancer (NSCLC) adenocarcinoma and pancreatic ductal adenocarcinoma (PDAC). Drug repurposing in oncology offers a cost-effective and time-efficient approach to developing new cancer therapies. Our research focuses on evaluating AF's selective cytotoxicity against cancer cells, identifying RNAseq-based biomarkers to predict AF response, and finding the most effective co-therapeutic agents for combination with AF.

**Methods:**

Our investigation employed a comprehensive drug screening of AF in combination with eleven anticancer agents in cancerous PDAC and NSCLC patient-derived organoids (*n* = 7), and non-cancerous pulmonary organoids (*n* = 2). Additionally, we conducted RNA sequencing to identify potential biomarkers for AF sensitivity and experimented with various drug combinations to optimize AF's therapeutic efficacy.

**Results:**

The results revealed that AF demonstrates a preferential cytotoxic effect on NSCLC and PDAC cancer cells at clinically relevant concentrations below 1 µM, sparing normal epithelial cells. We identified Carbonic Anhydrase 12 (CA12) as a significant RNAseq-based biomarker, closely associated with the NF-κB survival signaling pathway, which is crucial in cancer cell response to oxidative stress. Our findings suggest that cancer cells with low CA12 expression are more susceptible to AF treatment. Furthermore, the combination of AF with the AKT inhibitor MK2206 was found to be particularly effective, exhibiting potent and selective cytotoxic synergy, especially in tumor organoid models classified as intermediate responders to AF, without adverse effects on healthy organoids.

**Conclusion:**

Our research offers valuable insights into the use of AF for treating NSCLC and PDAC. It highlights AF's cancer cell selectivity, establishes CA12 as a predictive biomarker for AF sensitivity, and underscores the enhanced efficacy of AF when combined with MK2206 and other therapeutics. These findings pave the way for further exploration of AF in cancer treatment, particularly in identifying patient populations most likely to benefit from its use and in optimizing combination therapies for improved patient outcomes.

**Supplementary Information:**

The online version contains supplementary material available at 10.1186/s13046-024-03012-z.

## Background

Auranofin, originally approved as an oral gold-containing agent for the treatment of rheumatoid arthritis, has gained attention in the realm of drug repurposing for oncology. Its unique mechanisms of action, combined with its well-established safety profile, makes it an attractive candidate for potential therapeutic applications beyond its initial indication.

The biological mechanism of action is complex, but the redox enzymes thioredoxine reductase 1 (TrxR1) and TrxR2 are considered its main target. Gamberi et al. provide a clear overview of the repurposing potential of Auranofin as an innovative cancer therapy and the main downstream signaling pathways that are affected [1]. We have previously shown that high levels of mutant p53 protein sensitize non-small cell lung cancer (NSCLC) and pancreatic ductal adenocarcinoma (PDAC) cancer cells to AF and that AF triggers distinct molecular cell death mechanisms such as apoptosis and ferroptosis [2]. Despite this variety of downstream effects, cytotoxic effects could only be achieved at relatively high concentrations of Auranofin that are not achievable in patients. At a 6 mg/day dose of AF, the C_max_ levels of gold at day 7 was 1.57 μM (equivalent to 0.46 μM AF), and steady-state plasma gold concentrations following at least 12 weeks of 9 mg/day of AF were 1.0 ug/ml, corresponding to 1.5 μM AF [3, 4]. Ideally, efficacy with AF should be achieved at levels below 1 μM. An IC_50_ value below 1 μM was only achieved in one PDAC cell line (MIA PaCa-2) from a panel of 8 PDAC and NSCLC cell lines, and sensitivity was strongly related to high mutant p53 protein expression levels due to its inverse correlation with the expression of various antioxidants such as Nrf2, Trx and SOD1 [2]. These results indicate that AF as monotherapy would only be effective in a small subset of patients at clinically relevant concentrations. Consequently, combination strategies to enhance the therapeutic effect of AF are of high interest. Due to its broad activity, AF has been tested in combination with a wide variety of drugs, often leading to synergistic interactions. Camberi et al. summarized a range of combination strategies tested with AF, including, but not limited to, antioxidant inhibitors, PI3K/AKT/mTOR inhibitors, and various chemotherapeutics [1]. In addition, we have shown synergy with the PARP-1 inhibitor Olaparib in NSCLC and PDAC, independent of BRCA status, and cold-atmospheric plasma in glioblastoma, but only at higher concentrations [5, 6].

The goal of this study was to find the most potent combination strategy with AF to achieve high efficacy at clinically relevant concentrations. Therefore, we screened AF in combination with a panel of 11 drugs, including Anlotinib (multikinase inhibitor), Buparlisib (PI3Ki), MK2206 (AKTi), Everolimus (mTORi), Trametinib (MEK1/2i), ASTX029 (ERK1/2i), IM156 (OXPHOSi), Palbociclib (CDK4/6i) and the standard of care chemotherapeutics Cisplatin, Gemcitabine and Paclitaxel. The screening was performed on 4 NSCLC and 3 PDAC patient-derived organoids using an in-house developed live-cell imaging based organoid screening method. Image and data analysis were performed using the Orbits® platform [7, 8]. In addition, we have included two non-cancerous pulmonary organoids to investigate the selectivity of AF and the combination strategies towards epithelial NSCLC and PDAC cells. From this screening, we identified Carbonic Anhydrase 12 (CA12) as a strong RNAseq-based biomarker for AF sensitivity, probably due to its correlation with the NF-κB survival signaling pathway in response to oxidative stress. In addition, we show that the combination of AF with the AKT inhibitor MK2206 resulted in a strong synergistic cytotoxic effect, selective towards cancer cells.

## Methods

### Patient material

The patient-derived organoids used in this study are registered in the Biobank@UZA (Antwerp, Belgium; ID: BE71030031000); Belgian Virtual Tumorbank funded by the National Cancer Plan. Organoids were derived from resection fragments or biopsies obtained from cancer patients treated at the Antwerp University Hospital and a written informed consent was obtained from all patients. The study was approved by the UZA Ethical Committee (ref. 17/30/339 and 14/47/480). Lung cancer organoids (referenced by NKI_) were kindly provided by Emile E. Voest (Netherlands Cancer Institute) and obtained from the NKI-AVL biobank through a non-profit MTA [9]. An overview of the organoid lines is provided in Table S1 (Additional file 1).

### Organoid cultures

Basic medium consisted of Ad-DF +  +  + (Advanced DMEM/F12 (GIBCO), with 1% GlutaMAX (GIBCO), 1% HEPES (GIBCO), 1% penicillin/streptomycin (GIBCO) supplemented with 2% Primocin (Invivogen). For PDAC organoids, Ad-DF +  +  + was supplemented with 0.5 nM WNT Surrogate-Fc-Fusion protein (ImmunoPrecise), 4% Noggin-Fc Fusion Protein conditioned medium (ImmunoPrecise), 4% Rspo3-Fc Fusion Protein conditioned medium (ImmunoPrecise), 1 × B27 (Gibco), 10 mM nicotinamide (Sigma-Aldrich), 1.25 mM N-acetylcysteine (Sigma-Aldrich), 100 ng/ml FGF-10 (Peprotech), 500 nM A83-01 (Tocris), 10 nM gastrin (R&D Systems) and 10 μM Y-27632 after passaging (Selleck Chemicals). Normal pulmonary organoids and NSCLC organoids were cultured in Ad-DF +  +  + supplemented with 4% Noggin-Fc Fusion Protein conditioned medium (ImmunoPrecise), 4% Rspo3-Fc Fusion Protein conditioned medium (ImmunoPrecise), 1 × B27 (Gibco), 10 mM nicotinamide (Sigma-Aldrich), 1.25 mM N-acetylcysteine (Sigma-Aldrich), 100 ng/ml FGF-10 (Peprotech), 25 ng/ml FGF-7 (Peprotech), 500 nM A83-01 (Tocris) and 1 µM SB202190 (Sanbio, Cayman Chemical). For passaging, the organoids were digested to single cells with TrypLE Express (GIBCO) and resuspended in > 80% ice cold Cultrex growth factor reduced BME type 2 (R&D Systems) in full organoid medium. Small droplets of 20 µL were plated and were incubated inverted for 30 min at 37°C to allow them to solidify after which the drops were covered with full organoid medium. Characterization of the organoids used in this study has been described previously (normal pulmonary organoids [7], NSCLC [9] and PDAC [10]).

### RNA sequencing

For RNA sequencing (RNAseq), full grown organoids were harvested after 5 days of culture in ECM domes. Afterwards, RNA was extracted using RNeasy midi kit (Qiagen). For removal of gDNA, RNAse-free DNAse treatment was performed. RNA concentration and purity were checked using the Qubit RNA BR Assay Kit on Qubit 4 Fluorometer (ThermoFisher) and NanoDrop ND-1000 (ThermoFisher), respectively. Samples were frozen at -80 °C and delivered to Genomics Core Leuven for transcriptome sequencing using Lexogen QuantSeq 3’ FWD library preparation kit for Illumina on a Hiseq400 SR50 line with a minimum of 2M reads per sample. Downstream analysis and plotting (clustered heatmap, functional cluster annotation, enrichment analysis, partial correlation network, biomarker decision tree) were performed using the Omics Playground tool (Big Omics Analytics). For the predictive signatures, a genewise Pearson correlation was performed against the NOGR_AOC_1_fitted_n values of Auranofin (cut-off: *p* < 0.01).

### Drug screening

Drug screening on 3D organoids was performed at the DrugVision.AI automated screening facility of the University of Antwerp, Belgium, using a pre-validated drug screening pipeline for which a detailed protocol is available in the Journal of Visualized Experiments [8].

Briefly, established organoid lines were expanded in ECM domes (Cultrex type 2, Bio-Techne Ltd) in the absence of N-acetylcysteine and B27 supplements since they both have strong antioxidant properties. Instead, the medium was supplemented with N-2 (ThermoFisher Scientific) which contains among other selenites essential for an active thioredoxin system, the main target of AF. Next, 4-day-old organoids were harvested from ECM drops using the Cultrex Organoid Harvesting Solution (Bio-Techne Ltd), collected in a 15 mL tube coated with 0.1% BSA/PBS, washed, and resuspended in medium. Next, the number of organoids was quantified using imaging and diluted in full medium supplemented with 4% Cultrex at a concentration of 4000 organoids / mL. Next, 50 µL (200 organoids) of this solution was dispensed into each well of a 384-well ultra-low attachment microplate (Corning, #4588) using the OT-2 pipetting robot (Opentrons) in a cooled environment. Thereafter, the plate was centrifuged (100 rcf, 30 s, 4°C) and incubated overnight at 37°C. The following therapeutics were used: Auranofin, Anlotinib (multikinase inhibitor), Buparlisib (PI3Ki), MK2206 (AKTi), Everolimus (mTORi), Trametinib (MEK1/2i), ASTX029 (ERK1/2i), IM156 (OXPHOSi), Palbociclib (CDK4/6i) and the standard of care chemotherapeutics Cisplatin, Gemcitabine and Paclitaxel (Tocris Bioscience, MedChemExpress, Selleck Chemicals). All drugs and fluorescent reagents were added to the plate using the Tecan D300e Digital Dispenser and dissolved in either DMSO or 0.3% Tween-20 (Cisplatin). Cytotox Green (60 nM / well, Sartorius, DMSO) was used as a fluorescent cell death marker and Staurosporine (2 µM, Tocris Bioscience, DMSO) as a positive control. For each drug, a 6-point logarithmic titration was dispensed (10 – 5000 nM) in combination with a 4-point titration of Auranofin (500 nM—3000 nM) in a 6 × 4 synergy matrix using the Synergy tool of the D300e Control software. DMSO concentrations were normalized to the same level in each well (< 1%). Brightfield and green fluorescence whole-well images (4 × objective) were taken at 0, 72 and 120 h with the Tecan Spark Cyto set at 37°C / 5% CO2.

### Image and data analysis

Images and data were analysed with the Orbits® label-free organoid detection module [7]. Viability (V) was quantified as Total Brightfield Organoid Area – Total Green Area and excluding organoids that were classified as death by the label-free cell death detection module. V was used to calculate the Normalised Organoid Growth Rate (NOGR):

$$G=\frac{V\left(x\right)- V(0)}{V(0)}$$

$$if G>0: NOGR={G}_{drug}/{G}_{medNeg}$$

$$if G<0: NOGR={G}_{drug}/{G}_{medPos}$$

$$NOGR={\text{clip}}({\text{NOGR}}, [-1, 1])$$

where V(0) is the viability at timepoint 0, V(x) is the viability at timepoint x, G_drug_ is the G corresponding to the drug treated condition, G_medPos_ is the median G of the positive control and G_medNeg_ is the median G of the vehicle control.

Based on the NOGR, the drug effects can be classified as: > 1, proliferative effect; = 1, normal growth as in negative control; = 0, complete growth inhibition; = -1, complete killing as in positive control (Fig. S1, Additional file 1).

The dose–response relationship was modeled using the Growth Rate (GR) equation:

$$GR=GRinf+(1-GRinf)(\frac{1}{{1+\left(\frac{c}{GEC50}\right)}^{{h}_{GR}}})$$

where GRinf is the response at infinite concentration, GEC50 is the concentration that produces half the maximum possible effect, h_GR is the Hill coefficient, determining the steepness of the curve, and c is the concentration. Next, the Python SciPy library’s ‘curve-fit’ function was employed to fit the GR model to the observed data for each biological replicate. Initial guesses for GRinf, GEC50, and h_GR were set to 0.1, median concentration and 2, respectively. Residual errors between observed and predicted responses were calculated for each data point using the Root Mean Square Error approach. Points exhibiting an error greater than 2.5 times the mean error and an absolute error greater than 0.25 were deemed outliers and the model was refitted to this refined dataset. The following metrics were derived from the fitted curve: NOGR50 as the concentration at which the response is 0.5 and NOGR_AOC_1_fitted_n as the area over the curve (AOC) up to y = 1, normalized to the maximum area.

For synergy, a new derived variable Normalised (N)NOGR was computed to scale NOGR values between 0 and 100. The formula used for this computation is as follows:

$$NNOGR=\left(NOGR+1\right)* 50$$

The ZIP [11], Bliss [12], Loewe [13], Highest Single Agent (HSA) [14] synergy scores were calculated using the SynergyFinder R-package [15]. A synergy score > 10: Indicates a synergistic interaction between the drugs. -10 < Score < 10: Implies an additive effect where the combined impact of the drugs is approximately equal to their individual effects summed. < -10: Signifies an antagonistic interaction between the drugs.

To classify the cell lines into distinct response groups based on their NOGR_AOC_1_fitted_n values, a percentile-based approach was employed. The dataset was divided into three groups using the 33rd and 66th percentiles as boundaries: (1) Resistant: Cell lines with NOGR_AOC_1_fitted_nNOGR_AOC_1_fitted_n values below the 33rd percentile. (2) Intermediate: Cell lines with NOGR_AOC_1_fitted_n values between the 33rd and 66th percentiles. (3) Sensitive: Cell lines with NOGR_AOC_1_fitted_n values above the 66th percentile.

### Single-cell RNA sequencing public repositories and analysis

scRNA seq data for the PDAC dataset was obtained from GEO with accession number GSE205013 [16]. scRNA seq data for the NSCLC dataset was download from (https://doi.org/10.6084/m9.figshare.c.6222221.v3) [17]. In our study, we processed single-cell RNA sequencing data using Scanpy in Python. For the PDAC dataset, this involved filtering cells by minimum gene counts (200 genes) and genes by cell presence (in at least 3 cells). We identified mitochondrial, ribosomal, and hemoglobin genes for exclusion based on specific naming conventions and computed QC metrics to assess cell quality, including mitochondrial gene content. Outliers and cells with high mitochondrial content (> 20%) were removed. We further refined the dataset by normalizing gene expression levels to equalize sequencing depth across cells. We applied a logarithmic transformation to stabilize the variance across the data and scaled the data. Cancer cells were selected as KRT19 + or EPCAM + cells for further downstream analysis. For the NSCLC dataset, the Seurat object was converted to Scanpy compatible H5AD files in R. This dataset included extensive annotation and was filter for Subtype ‘adenocarcinoma’ and Cell_Cluster_level1 ‘Cancer’ to select for cancer cells. Data scaling and dimensionality reduction through PCA and UMAP were performed to visualize and interpret the complex dataset effectively for both datasets filtered for cancer cells. Spearman correlation between the percentage of CA12 and NFKB1, NFKB2, RELA and RELB positive cells per patient was performed and a *p*-value < 0.05 indicates a significant correlation.

## Results

### High-throughput drug screening

A total of 18 384-well micro-well plates were used in this study, two for each organoid line, on which 11 drug combinations were dispensed in a 4 × 6 drug synergy matrix (AF x DrugX).

**Fig. 1:**
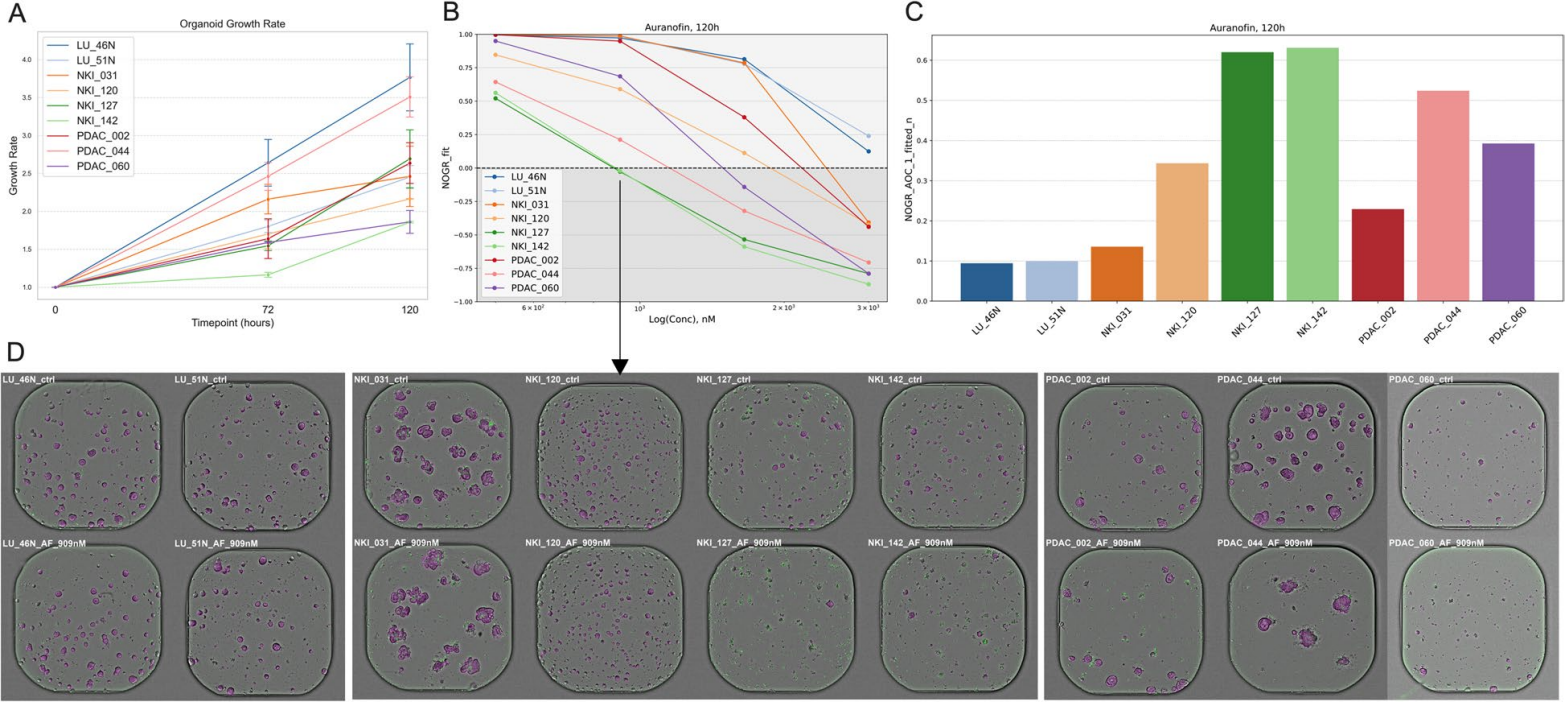
Auranofin monotherapy. **A** Image-based quantification of organoid growth rate based on the Viability (V) metric, normalized to timepoint 0 (mean ± SD, *n* = 2). **B** Fitted dose–response curves of the mean (*n* = 2) Normalised Organoid Growth Rate (NOGR) metric for the treatment with Auranofin (500, 909, 1651, 3000 nM) for 120 h. **C** Normalized Area Over the Curve (AOC) values of the fitted NOGR dose–response curves following Auranofin treatment. **D** Representative images of organoids treated with vehicle (DMSO) or 909 nM Auranofin for 120 h. Magenta = label-free organoid segmentation by Orbits®; Green = raw cytotox green signal; LU_ = normal pulmonary organoids; NKI_ = lung cancer organoids; PDAC_ = pancreatic ductal adenocarcinoma organoids

The growth rate of different organoid lines was measured over 120 h using image-based quantification. It was clear that baseline growth rate varied significantly across the different organoid lines (Fig. 1A), thus highlighting its importance when evaluating the effects of drugs and drug combinations on organoids. Therefore, we employed a growth rate-based metric to evaluate therapeutic effect, the normalized-organoid growth rate (NOGR), which considers basal organoid growth rate as well as viability (Fig. S1, Additional file 1). At day five, 5472 unique NOGR datapoints were obtained for all 9 lines of which 147 (2.69%) were identified as outliers in relation to the fitted drug-response curve and excluded from further analysis. A high correlation was observed between the non-fitted and fitted NOGR values (*r* = 0.94) (Fig. S2, Additional file 1). Fitted NOGR values were used for further downstream analysis.

### AF is selective towards NSCLC and PDAC organoids compared to pulmonary organoids

Organoids were treated with 4 concentrations of AF (500, 909, 1651, 3000 nM) for 120 h and a strong variability in response was observed between the patients (Fig. 1B-C). Notably, the normal pulmonary organoids, specifically LU_46N and LU_51N, demonstrated the highest resistance. They showed no measurable response to AF at concentrations below 1000 nM. Figure 1D presents images of organoids treated with 909 nM AF. These images highlight the precision of the NOGR metric we utilized to categorize the cytostatic effect of the drug at this concentration, where 1 > NOGR > 0. Cytotoxicity could only be achieved in certain tumor organoid lines at higher concentrations of AF, which are more challenging to achieve in the patient as discussed above (> 1500 nM). This highlights the need for combination strategies to enhance the efficacy of AF.

### Low CA12 expression is associated with high AF sensitivity

**Fig. 2:**
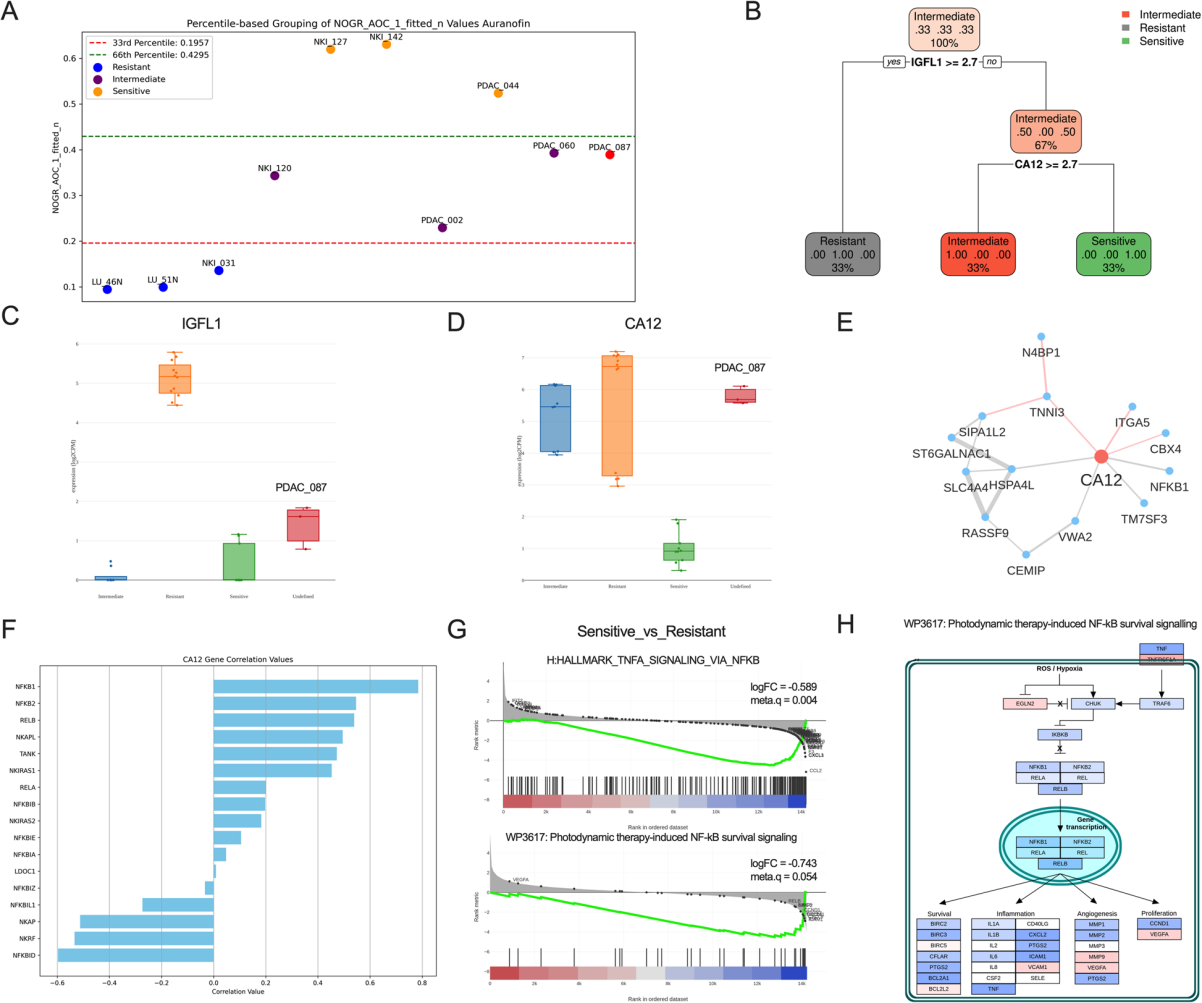
Predictive biomarkers for Auranofin response. **A **Percentile-based classification of Auranofin response into sensitive, intermediate and resistant groups. **B** Decision tree-based classification of the 3 response groups based on the topmost important features. PDAC_087 was excluded from this analysis to be used as a testing sample. Log2(counts per million reads, CPM) expression values for IGFL1 (**C**) and CA12 (**D**). **E** Partial correlation network for CA12. Grey edges correspond to positive correlation, red edges to negative correlation. The width of the edge is proportional to the absolute partial correlation value of the gene pair. **F** Positive and negative correlation of CA12 expression with members of the NF-kB signaling pathway. **G** Gene set enrichment analysis of NF-kB signaling related genesets, following differential expression analysis between the sensitive and resistant organoids. **H** Up- (red) and down- (blue) regulated genes of the NF-kB survival signaling pathway in the resistant versus sensitive organoids

**Fig. 3:**
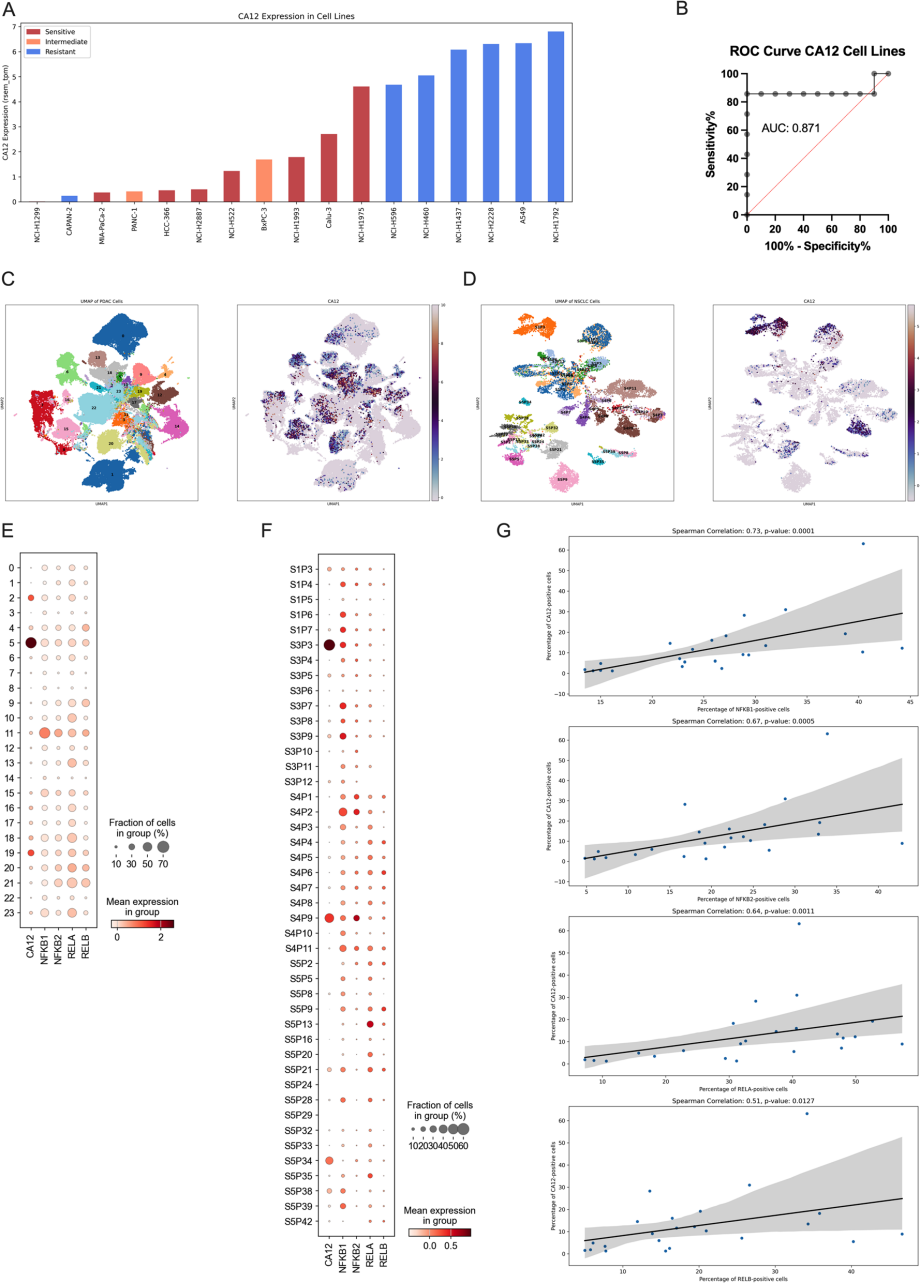
CA12 as predictive biomarker in 2D cancer cell lines and expression in patient samples.** A** CA12 expression values (transcript per million) derived from a publicly available RNA-Seq dataset for 17 2D NSCLC and PDAC cancer cells lines for which the AF treatment response was defined in previous studies. **B** ROC analysis for the classification of resistant and sensitive (sensitive + intermediate) cell lines to AF treatment, based on CA12 expression values. UMAP overview of cancer cells annotated by patient and CA12 expression for (**C**) PDAC and **D** NSCLC adenocarcinoma patient samples. Dotplot representing the fraction of positive cells (%) and mean expression per patient for (**E**) PDAC and (**F**) NSCLC patients. **G** Scatter plot visualising the correlation between the percentage PDAC positive cells for CA12 and NFKB-related genes. The Spearman correlation coefficient and related *p*-value is plotted. (*p* < 0.05 indicates significance)

We classified the 9 organoid lines in resistant, intermediate, and sensitive based on the normalized area over the curve (AOC) of the fitted NOGR dose–response curve (Fig. 2A). A higher value indicates a stronger response. Using this classification, we performed a biomarker analysis on the baseline transcriptome of the organoids, which ranked high IGFL1 expression as the strongest biomarker for resistance and low CA12 expression as the strongest biomarker for sensitivity (Fig. 2B). Next, we tested this decision tree on a new PDAC organoid line PDAC_087, with low IGFL2 expression and high CA12 expression (Fig. 2C-D). Based on these markers, we correctly classified PDAC_087 as an intermediate responder, corresponding to its NOGR AOC value below 0.4295 (Fig. 2A). From the partial correlation network for CA12, we identified a positive correlation with NFKB1. Since AF is known to inhibit NF-κB signaling, we performed a correlation analysis of CA12 and NF-κB related genes and found a strong positive correlation with among others the NKFB1, NFKB2 and RELB subunits and a negative correlation with NFKBID and NKRF, which are negative regulators of NF-κB signaling (Fig. 2F). Furthermore, gene sets related to NF-κB signaling were negatively enriched in the sensitive versus resistant organoids, including the NF-κB survival signaling in response to ROS/Hypoxia (Fig. 2G-H). Consequently, cells with impaired NF-κB signaling are more sensitive to AF treatment. Although we are the first to screen AF in NSCLC and PDAC organoids, we and Li et al. have already screened AF in a combined panel of 17 2D cancer cell lines [2, 18]. By combining the AF response classification with publicly available RNA-Seq data from these cell lines [19], we show that all but one resistant cell lines have high expression levels of CA12 (Fig. 3A). ROC analysis further supported the use of CA12 as an accurate biomarker for AF response (sensitive vs. resistant) in these cell lines (AUC: 0.871, *p*-value: 0.0112) with a sensitivity of 86% and specificity of 100% at a TPM cut-off of 4.645 (Fig. 3B). Based on the comprehensive analysis of organoid and 2D cancer cell line responses to AF, we show that low CA12 expression is a reliable biomarker for predicting sensitivity to AF treatment. To examine the clinical significance of CA12, we analyzed its expression using single-cell RNA sequencing data from publicly accessible databases, focusing on PDAC with 23 patient samples and NSCLC adenocarcinoma with 44 patient samples, specifically within cancer cell populations [11, 12]. In line with our observations in PDOs, we noted a pronounced heterogeneity in CA12 expression among patients, which was also evident at the level of individual patients between cells (Fig. 3C-F). Expression was generally higher in PDAC patients compared to NSCLC patient. Notably, the proportion of CA12-expressing cancer cells within a patient exhibited a strong positive correlation with key components of the NF-κB signaling pathway — NFKB1 (*r* = 0.73, *p* < 0.0001), NFKB2 (*r* = 0.67, *p* < 0.0005), RELA (*r* = 0.64, *p* < 0.011), and RELB (*r* = 0.51, *p *< 0.0127) — in PDAC samples (Fig. 3G). This pattern was not replicated in NSCLC samples (Fig. S3, Additional file 1), possibly due to their lower overall expression levels of CA12. Additionally, the quality of the PDAC dataset was markedly superior to that of the NSCLC dataset, which necessitates caution when drawing definitive conclusions from the latter (Fig. S4, Additional file 1).

### Predictive transcriptome signatures

**Fig. 4:**
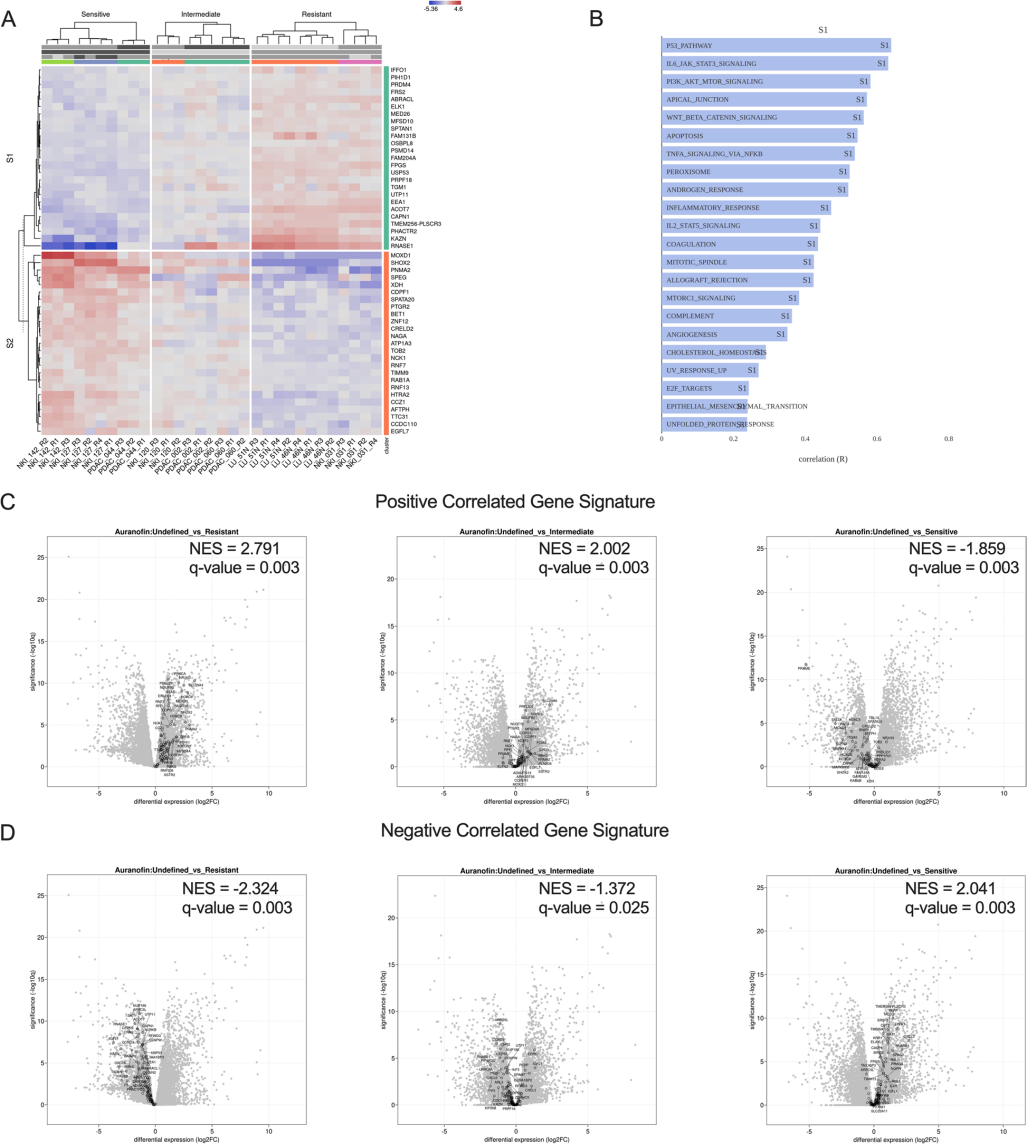
Predictive signatures for Auranofin response.** A** Clustered heatmap of the top 25 negative (cluster S1) and top 25 positive (cluster S2) correlated genes with the normalized Area Over the Curve (AOC) values of the fitted dose–response curves for Auranofin in 9 organoid lines, excluding PDAC_087. **B** Functional annotation of cluster S1 for the Hallmark geneset collection. **C-D** Volcano plots and normalized enrichment score (NES) of the predictive signatures derived from significantly (*p* < 0.01) positive (*n* = 86) and negative correlated genes (*n* = 128) for PDAC_087 versus the sensitive, intermediate and resistant organoids

Besides looking at a single biomarker, we aimed to make predictive transcriptome signatures based on the top correlated genes with the AF NOGR AOC values of the 9 organoid lines. The heatmap in Fig. 4A shows the top 25 negatively (S1) and top 25 positively (S2) correlated genes with the corresponding functional annotation to the Hallmark gene set collection of cluster S1. Interestingly, samples with high p53 pathway activity, IL6_JAK_STAT3 and PI3K_AKT_MTOR signaling appeared to be more resistant to AF, which is in line with previous findings and support the biological relevance of our approach (Fig. 4B). Next, we made two predictive signatures from significantly (*p* < 0.01) positive correlated genes (*n* = 86) and negative correlated genes (*n* = 128) and performed gene set enrichment analysis for PDAC_087 versus the Sensitive, Intermediate and Resistant organoids to determine if we could correctly classify PDAC_087 as an intermediate responder based on our signatures. Compared to the resistant group, PDAC_087 was positively enriched (NES = 2.791, *p* = 0.003) for genes in the positive correlated gene signature (PCGS) and negatively enriched (NES = -2.324, *p* = 0.003) for genes in the negative correlated gene set (NCGS) indicating that PDAC_087 will respond to AF (Fig. 4C). In contrast, PDAC_087 was negatively enriched (NES = -1.859, *p* = 0.003) for genes in the PCGS compared to the sensitive group, and positively enriched (NES = 2.041, *p* = 0.003) for genes in the NCGS, indicating that it has an intermediate response (Fig. 4D). These results successfully demonstrate the utility of predictive transcriptome signatures in classifying organoids into appropriate AF response categories.

### Synergy

The objective of our study was to evaluate the effectiveness of AF when used together with eleven different anticancer drugs. For this purpose, we administered each of these drugs in a six-level logarithmic concentration range from 10 to 5000 nM, in combination with AF administered in a four-level concentration range from 500 to 3000 nM. This approach created a synergy matrix of 6 × 4, allowing for an extensive assessment of the combined effects of AF and each anticancer drug. For every combination within this matrix, we quantified the NOGR values. This measurement enabled us to evaluate both the cytostatic (cell growth-inhibiting) and cytotoxic (cell-killing) effects from live-cell imaging as detailed in the materials and methods section and visualized in Figure S1 (Additional file 1). By analyzing these data, we were able to identify the combinations that most effectively induced cell death, pointing to the most promising strategies for combination therapy involving AF and these anticancer drugs. In order to thoroughly determine the degree of combination synergy and select the best model for our study, we compared the commonly-used synergy models HSA, Loewe, Bliss, and ZIP. The results from ZIP and Bliss showed a strong Pearson correlation (*r* = 0.96), suggesting similar outcomes (Fig. S5A, Additional file 1). However, the conclusions drawn from these models compared to HSA and Loewe varied significantly (Fig. S5B-F, Additional file 1). For instance, the combination of AF with Everolimus was identified as synergistic by the HSA and Loewe models, but only additive by the Bliss and ZIP models in the NKI-120 context. Notably, several combinations did not meet the basic synergy concept (1 + 1 > 2), as illustrated in Fig. S5G-I (Additional file 1). Based on its stringency and accuracy in quantifying true synergism, we selected the ZIP model as the most appropriate for our analysis.

**Fig. 5:**
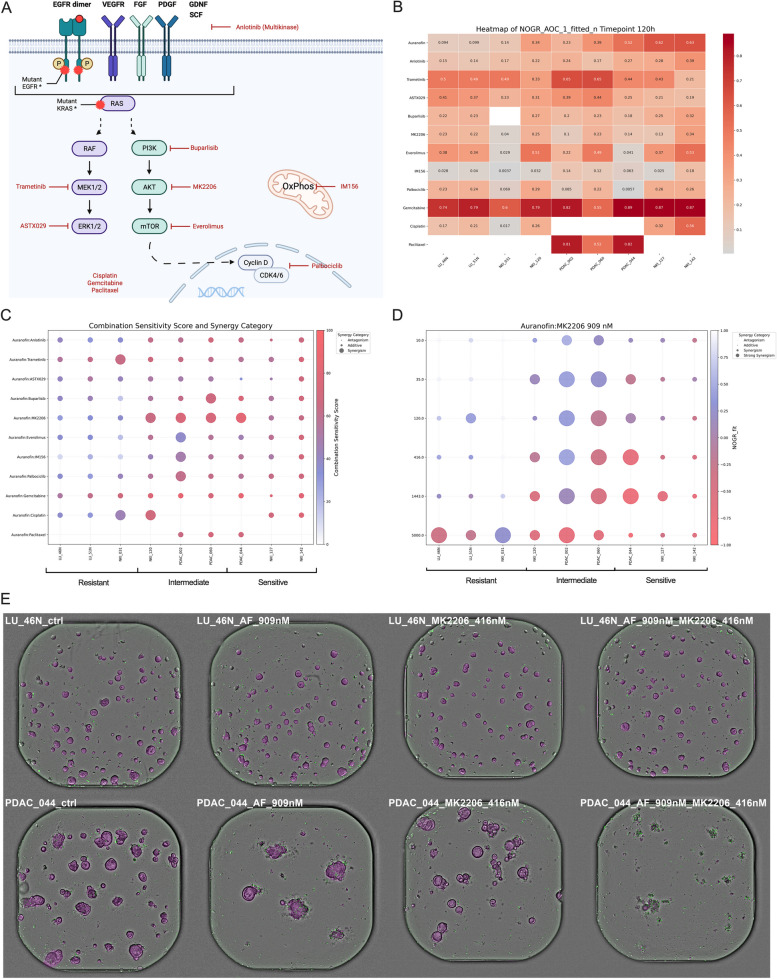
Auranofin drug combination strategies.** A** Overview of the 11 therapeutics that were tested in combination with Auranofin. **B** Heatmap showing the normalized Area Over the Curve (AOC) values of the fitted Normalised Organoid Growth Rate (NOGR) dose–response curves for each therapy and organoid line. **C** Bubble plot showing the mean ZIP synergy score (bubble size) and combination sensitivity score (CSS, colored heatmap) for each drug and organoid line. **D** Bubble plot showing the ZIP synergy score (bubble size) and NOGR (colored heatmap) for a concentration range of MK2206 and 909 nM Auranofin for each organoid line. Bubble size: small = ZIP < -10 indicating antagonism; medium = -10 < ZIP < 10 indicating an additive effect; large = ZIP > 10 indicating synergism. NOGR between 1 and 0 indicates a cytostatic response, NOGR < 0 indicates a cytotoxic response. The resistant, intermediate and sensitive groups refer to the Auranofin response classification. **E** Representative images of organoids (PDAC_044) treated with vehicle (DMSO), 416 nM MK2206, 909 nM Auranofin or the combination for 120 h. Magenta = label-free organoid segmentation by Orbits®; Green = raw cytotox green signal; LU_46N = normal pulmonary organoid; PDAC_044 = pancreatic ductal adenocarcinoma organoid

Figure 5A presents the targets for the 11 compounds we selected for our combination screening, and Fig. 5B lists the normalized AOC values of the fitted NOGR curves for each monotherapy across all organoid lines. Notably, AF emerged as the only compound demonstrating selectivity for cancer cells over healthy pulmonary cells. The most pronounced synergistic and cytotoxic effect, as indicated by a high combination sensitivity score (CSS), was observed in combination with the AKT inhibitor MK2206 (Fig. 5C). This effect was particularly significant in the group characterized as intermediate responders to AF and absent in the healthy organoids (Fig. 5D-E). In the highly sensitive organoids, an additive effect was still obtained, resulting in high cell death of the organoids at low concentrations of MK2206 and AF (909 nM) (Fig. 5D), making this a highly potent combination strategy. Interestingly, this was not observed when blocking the PI3K-AKT-MTOR axis upstream (Buparlisib, Fig. S6A, Additional file 1) or downstream (Everolimus, Fig. S6B, Additional file 1) of AKT.

**Fig. 6:**
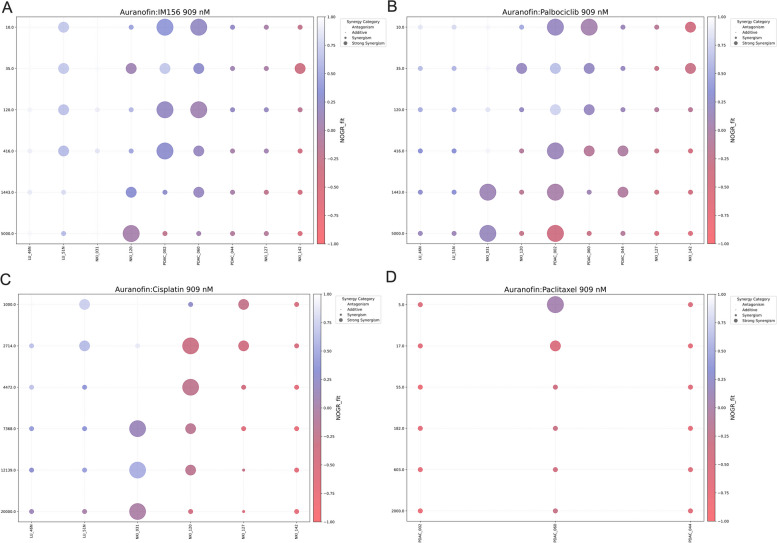
Selected Auranofin drug combination strategies. Bubble plots showing the ZIP synergy score (bubble size) and Normalized Organoid Growth Rate (NOGR, colored heatmap) for a concentration range of **A** IM156, **B** Palbociclib, **C** Cisplatin and **D** Paclitaxel in combination with 909 nM Auranofin for each organoid line. Bubble size: very small = ZIP < -10 indicating antagonism; small = -10 < ZIP < 10 indicating an additive effect; medium = 10 < ZIP < 20 indicating moderate synergism; large = ZIP > 20 indicating strong synergism. NOGR between 1 and 0 indicates a cytostatic response, NOGR < 0 indicates a cytotoxic response

Investigating the MAPK/ERK pathway, we found that Trametinib (MEK1/2) and ASTX029 (ERK1/2) did not selectively target cancer cells. This indicates that the MAPK/ERK pathway may be essential for the survival of normal epithelial cells, at least in organoids (Fig. 5B). Trametinib showed cytotoxic synergy with 909 nM AF in some tumor organoids at higher concentrations, but this was not selective for cancer cells (Fig. S6C, Additional file 1). Similarly, ASTX-029 demonstrated only a few instances of synergistic interactions (Fig. S6D, Additional file 1), akin to the multi-kinase inhibitor Anlotinib (Fig. S6E, Additional file 1). For IM156 and Palbociclib, a moderate to strong selective synergistic effect was observed when combined with 909nM AF in PDAC_002 and PDAC_060, both intermediate AF responders. However, it's important to note that this synergy was predominantly cytostatic, failing to induce cell death in cancer cells within the nanomolar range (Fig. 6A-B).

Finally, a selection of standard of care chemotherapy agents showed moderate to strong cytotoxic synergy when combined with Cisplatin, particularly in the Cisplatin resistant NKI_031 and intermediate responders NKI_120 and NKI_127 (Fig. 6C, Fig. 4B) Notably, this synergistic effect was not observed in the organoid line that exhibited the strongest response to Cisplatin (NKI_142, Fig. 5B). In a similar pattern, synergy with Paclitaxel was observed exclusively in PDAC_060, which was the least responsive to Paclitaxel (Fig. 6D, Fig. 5B). While the concentration range for Gemcitabine was suboptimal due to its strong cytotoxic effect as a monotherapy, a notable observation was made at its lowest concentration. Here, a moderate synergistic effect with 909 nM AF was detected in the most resistant organoids, NKI_031 and PDAC_060, as shown in Figure S6F-G (Additional file 1).

## Discussion

In this study, we aimed to address three critical aspects related to the use of Auranofin in the treatment of PDAC and NSCLC: (1) the selectivity of AF towards cancer cells in relation to normal epithelial cells, (2) the identification of RNAseq-based predictive biomarkers and signatures, and (3) the identification of the most effective co-therapeutic agent that exhibits strong cytotoxic synergy, with a selective action towards cancer cells. To achieve these objectives, we performed a synergy screening of 11 therapeutic agents on normal pulmonary, PDAC and NSCLC organoids. This innovative approach allowed us to directly compare the efficacy of different combination strategies, advancing our understanding of treatment interactions with AF.

In relation to selectivity, we show that the viability and growth of normal epithelial cells was unaffected by AF at clinically achievable concentrations below 1 μM, while cytostatic efficacy was observed in five out of seven tumor organoids. However, toxicity of AF remains an important aspect to be considered. In mice, we have previously shown strong gut-related toxicity, discomfort and weight loss following daily intraperitoneal injection of 10 mg/kg AF. Following the clinical administration route, AF was well tolerated through oral gavage but showed only limited antitumoral efficacy [20]. In this manuscript we also provide a clear overview of all the in vivo studies that were performed with AF for the treatment of cancer. In adult RA patients, the optimal long-term dosage of AF is 6 mg per day, either as a single dose or split doses. Clinical trials involving over 5,000 RA patients, some treated for more than 7 years, monitored AF's safety and efficacy. Generally, AF showed no significant cumulative toxicity in RA patients. Adverse events during AF treatment are usually mild, transient, and often resolve with ongoing treatment or dose reduction. These events mostly occur in the initial months of therapy, with their frequency decreasing over time [21]. As mentioned in the introduction, 6 mg/day dose of AF reached C_max_ levels equivalent to 0.46 μM AF, which could be increased to 1.5 μM AF following at least 12 weeks of 9 mg/day [3, 4]. To apply AF as anticancer therapy, it will be crucial to identify patients that are sensitive to AF at clinically achievable levels below 1 μM. In our study, we have identified low CA12 levels as a strong biomarker for response and validated its predictive value in publicly available cell line datasets [2, 18, 19]. Our observations suggest a link between high levels of CA12 and increased NF-κB activity in epithelial cancer cells. This is evidenced by the fact that cancer cells with low CA12 levels display weakened NF-κB survival signaling, as shown by the negative enrichment of NF-κB -related gene sets in organoid lines sensitive to AF and with low CA12 expression. Interestingly, Du et al.'s research shows that PDAC patients with lower CA12 expression had significantly reduced overall survival compared to those with higher CA12 expression, with a Hazard Ratio (HR) of 2.724 [22]. Consequently, patients with the worst outcome could benefit the most from AF treatment based on our proposed biomarker. In contrast, they also found that overexpression of CA12 led to decreased levels of NF-κB p65 (RELA) and increased levels of IκBα (NFKBIA), suggesting an inhibition of the canonical NF-κB complex (p65/p50 dimer) by CA12. However, our data show a different aspect: CA12 mRNA expression levels correlate positively with the mRNA levels of the non-canonical NF-κB subunits p52 (NFKB2) and RelB (RELB). This indicates that CA12 might influence the non-canonical NF-κB signaling pathway, which activates the RelB/p52 NF-κB complex, known for more sustained activity in cells compared to the canonical pathway [22]. This requires more in-depth research at the protein level and falls outside the scope of this study. AF is notably effective in inhibiting NF-κB signaling, a pathway crucial for cellular antioxidant responses [23]. Research by Jeon et al. demonstrated that AF doses between 5–10 µM block IκB kinase (IKK), essential for NF-κB activation [24, 25]. Saei et al. identified NF-κB as a target of AF at a 3 µM concentration in colon cancer cells [26]. Further, Nakaya et al. found that even at 0.05 µM, AF inhibits NF-κB DNA binding and reduces its nuclear protein levels [27]. Given the fact that high NF-κB activity was related to resistance to AF in our study in the normal pulmonary organoids, we would advise against combination strategies with NF-κB inhibitors since this might be an important protection mechanism in non-cancerous cells.

To increase the efficacy of AF at clinically achievable concentrations, combination strategies are a promising strategy and various combinations have been tested in vitro and in vivo [1, 20]. In our study it was clear that the AKT inhibitor MK2206 was the most potent co-therapeutic for AF. This combination resulted in a selective, robust, and synergistic cytotoxic effect, significantly surpassing the efficacy of other tested therapeutics in nearly all tumor organoid models. Dai et al. made a similar observation and identified that inhibition of TXNRD1 with siRNAs or AF sensitized lung cancer cells to MK2206 in vitro and in vivo (H1993) [28]. Single drug treatment had no effect on tumor growth, while the combination resulted in a remarkable 85% reduction of tumor volume compared to each drug alone, prolonging the survival of mice from 31 to 76 days without any reported toxicity issues. Further, Li et al. also concluded that TXNRD participates in the regulation of the PI3K/AKT/mTOR pathway, and that AF inhibits the expression and/or phosphorylation of multiple key nodes in the pathway [18]. Consequently, AF is being investigated in combination with the mTOR inhibitor Sirolimus in lung cancer (NCT01737502) and ovarian cancer (NCT03456700). In the latter, it became clear that toxicity of this combination strategy is of concern since serious and other adverse events were detected in 42% and 95% of the patients, respectively, after receiving 6 mg AF and 5 mg Sirolimus once daily on days 1 to 28. Importantly, no confirmed tumor response was observed. In our study, we observed limited synergy with Everolimus, a derivative of Sirolimus, which is more in line with the clinical observations. In contrast, Xia et al. report a strong synergy between Everolimus and AF in colorectal (HCT116) and gastric cancer (SGC-7901) xenografts in nude mice without weight loss or other signs of toxicity [29].

In our study, we verified earlier research showing that AF enhances the effectiveness of Cisplatin, Paclitaxel, and Gemcitabine [30–32]. This was observed through increased synergistic cytostatic/cytotoxic effects in tumor organoid lines that initially exhibited greater resistance to these chemotherapy drugs. For Palbociclib, Kratzke et al. previously reported a synergistic effect decreasing cell proliferation in two mesothelioma cell lines H2373 and H2452 [33]. In our study we also observed selective and moderate to strong synergy in several tumor organoid models, although the effect was mainly cytostatic. Similarly, the combination with IM156 yielded moderate to strong synergistic effects but resulted in only a limited antitumoral effect in the cytostatic range.

## Conclusions

In conclusion, our study provides significant insights into the potential of AF as a therapeutic agent for PDAC and NSCLC adenocarcinoma. We demonstrated that AF exhibits selectivity towards cancer cells at clinically achievable concentrations below 1 μM, with minimal impact on normal epithelial cells. Crucially, our identification of low CA12 levels as a predictive biomarker for AF response offers a promising avenue for personalized cancer therapy. The robust synergy observed with the AKT inhibitor MK2206 highlights the effectiveness of combination strategies in enhancing AF's anticancer potential. Our findings pave the way for further exploration of AF in cancer treatment, particularly in identifying patient populations most likely to benefit from its use and optimizing combination therapies for improved outcomes.

### Supplementary Information

**Supplementary Material 1. **

## Data Availability

The data that support the findings of this study are available from the corresponding authors upon reasonable academic request.

## Abbreviations

AF: Auranofin
AKT: Protein Kinase B (also known as PKB)
AOC: Area Over the Curve
CA12: Carbonic Anhydrase 12
CSS: Combination Synergy Score
ERK1/2: Extracellular Signal-Regulated Kinases 1/2
HSA: Highest Single Agent
MEK1/2: Mitogen-Activated Protein Kinase Kinase 1/2
mTOR: Mammalian Target of Rapamycin
NKI: Netherlands Cancer Institute
NOGR: Normalised Organoid Growth Rate
NSCLC: Non-small Cell Lung Cancer,
OXPHOS: Oxidative Phosphorylation
PDAC: Pancreatic Ductal Adenocarcinoma
PI3K: Phosphoinositide 3-Kinase
UZA: Antwerp University Hospital
