# Supplementary material for: Auranofin repurposing for lung and pancreatic cancer: low CA12 expression as a marker of sensitivity in patient-derived organoids, with potentiated efficacy by AKT inhibition
Source: J Exp Clin Cancer Res. 2024 Mar 22;43:88. doi: 10.1186/s13046-024-03012-z (PMC10958863; doi:10.1186/s13046-024-03012-z)
Supplement: Supplementary file 1 — Supplementary Material 1. [file 13046_2024_3012_MOESM1_ESM.pdf]

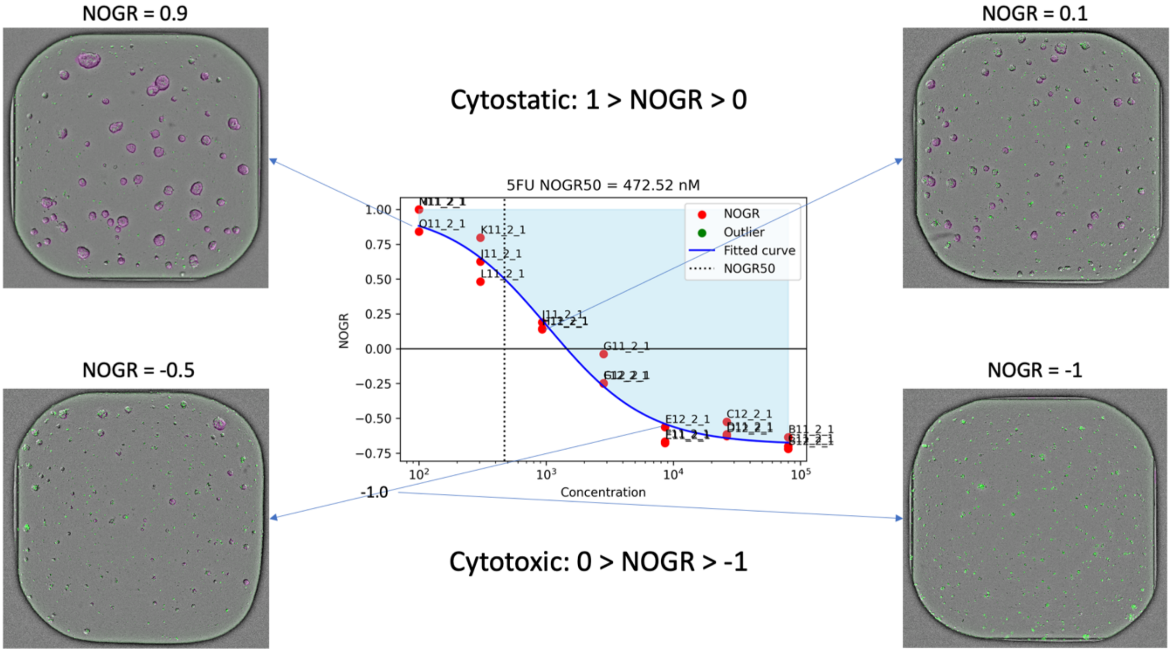

Supplemental Figure 1: Visualisation and interpretation of the Normalised Organoid Growth Rate (NOGR) drug response metric of PDAC organoids treated with a concentration range of 5-fluoro-uracil (5-FU). Magenta indicates label-free organoid segmenation from brightfield images using Orbits® and green indicates raw green fluorescence signal of the cytotoxic green cell death marker. An NOGR between 1 and 0 indicates a cytostatic effect (growth arrest) and a value between 0 and-1 a cytotoxic effect (cell death).

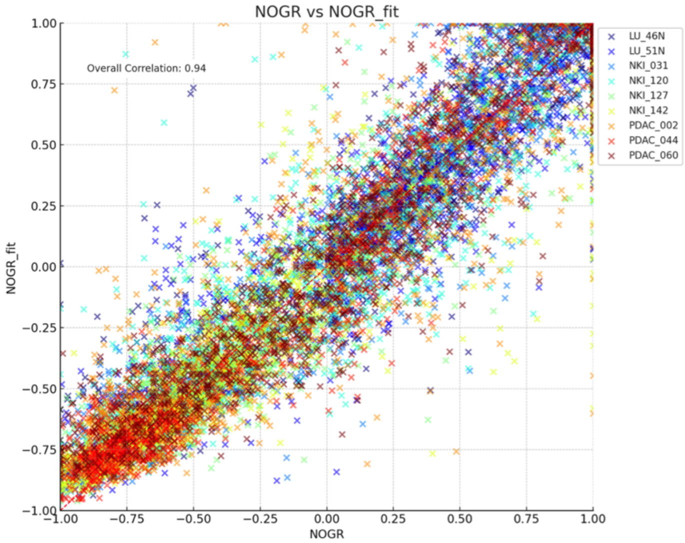

Supplemental Figure 2: Scatterplot of the fitted Normalized Organoid Growht Rate (NOGR) value versus the measured NOGR values. The overall correlation represents the Pearson correlation value.

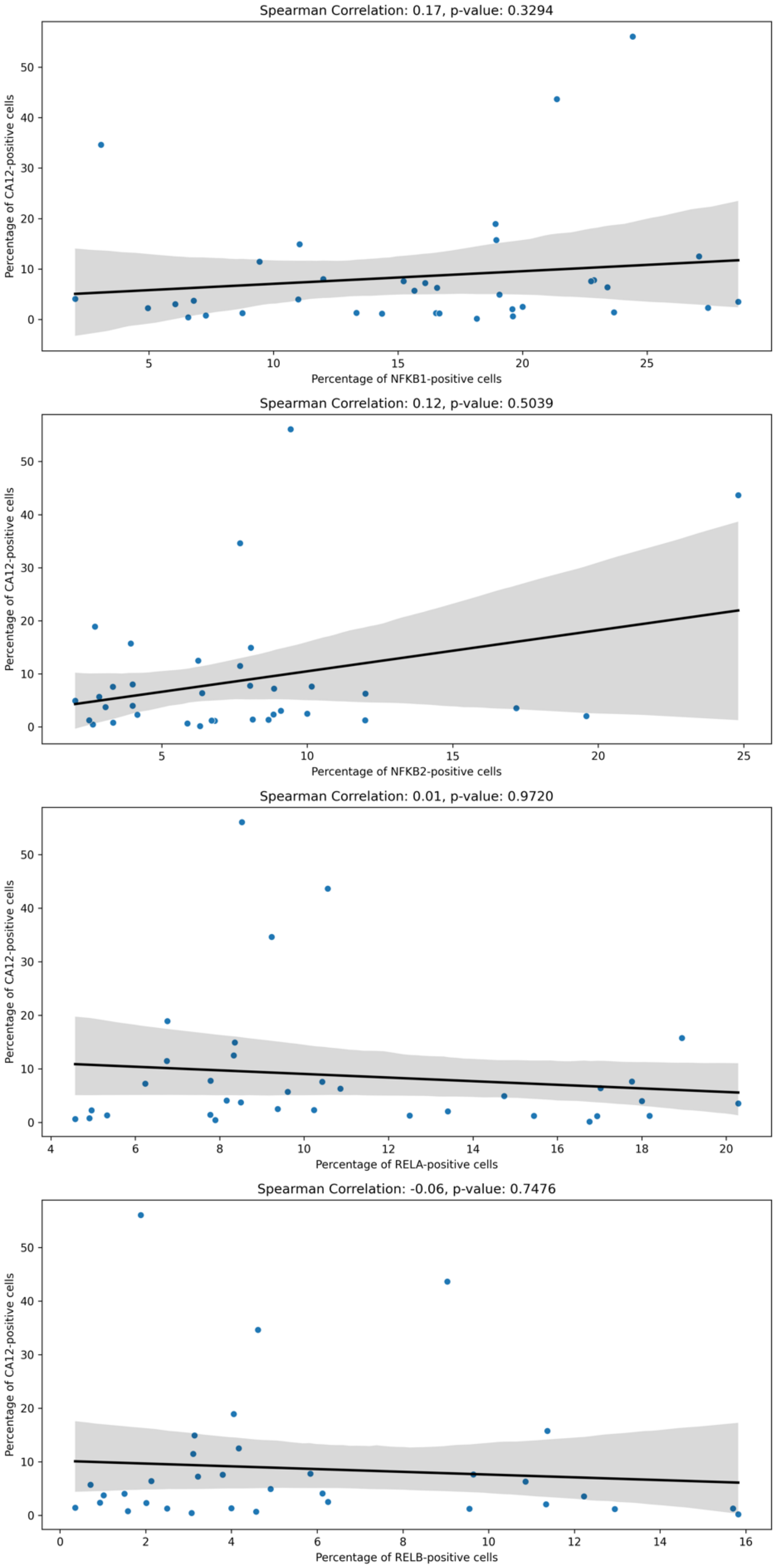

Supplemental Figure 3: Scatter plot visualising the correlation between the percentage NSCLC positive cells for CA12 and NFKB-related genes. The Spearman correlation coefficient and related p-value is plotted. ($p < 0.05$ indicates significance).

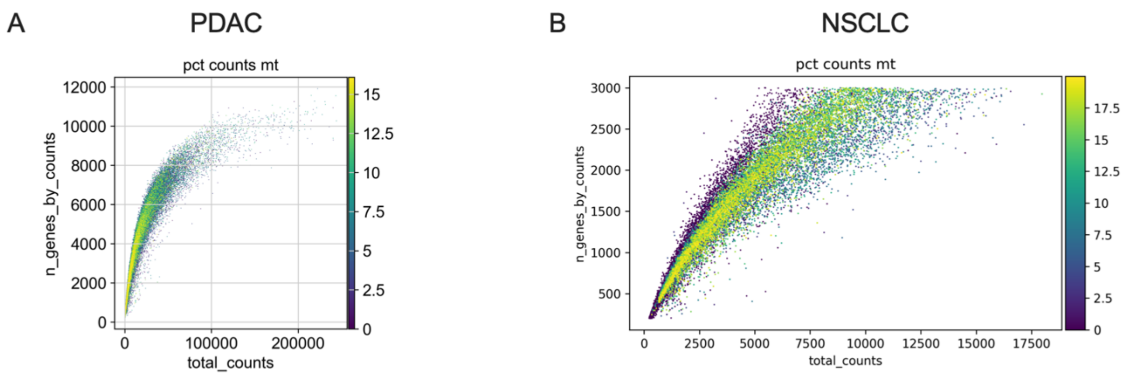

Supplemental Figure 4: This scatter plot visualizes key quality control metrics after quality control and filtering for the (A) pancreatic ductal adenocarcinoma and (B) non-small cell lung cancer adenocarcinoma scRNA-seq datasets. The x-axis ('total_counts') represents the total number of transcript counts detected in each cell, indicating the overall library size or sequencing depth per cell. The y-axis ('n_genes_by_counts') shows the number of unique genes detected with at least one count in each cell, serving as a measure of the cell's transcriptomic complexity. Cells are colored according to 'pct_counts_mt', the percentage of mitochondrial gene counts out of the total counts, which is depicted by the color gradient. A higher percentage of mitochondrial counts is often associated with cell stress or apoptosis, as well as potential technical artifacts. A cut-off of 20% mitochondrial gene content was applied.

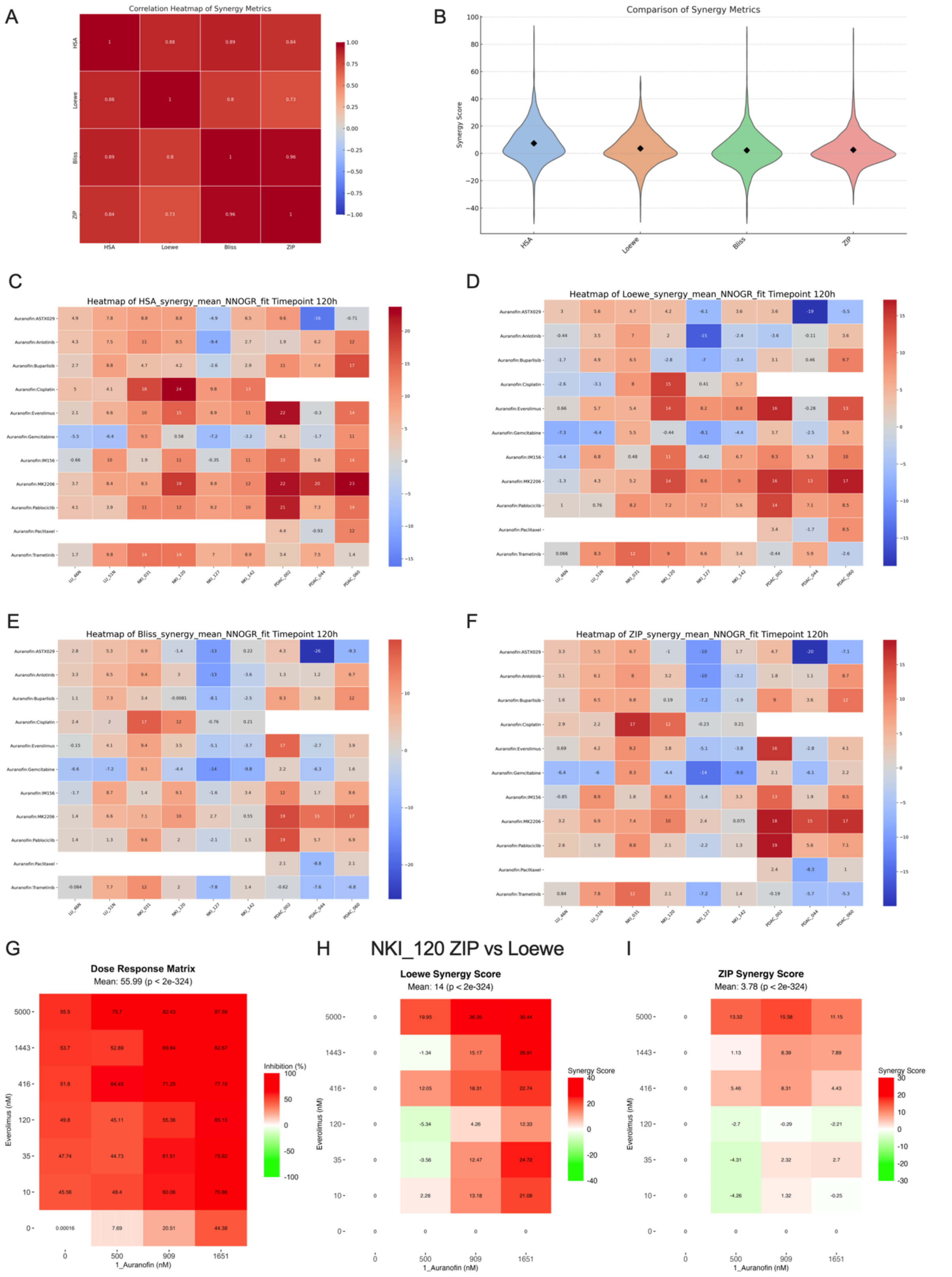

Supplemental Figure 5: Comparison of synergy models. (A) Pearson correlation heatmap for HSA, Loewe, Bliss and ZIP synergy models. **(B)** Violin plots for the mean synergy scores of each model. (C-F) Heatmap of the mean synergy score of all the drug combinations for each organoid lines. (G) Dose response matrix showing the NNOGR values for the combination of Auranofin with Everolimus in NKI_120. (H-I) Corresponding synergy scores for the Loewe and ZIP synergy model.

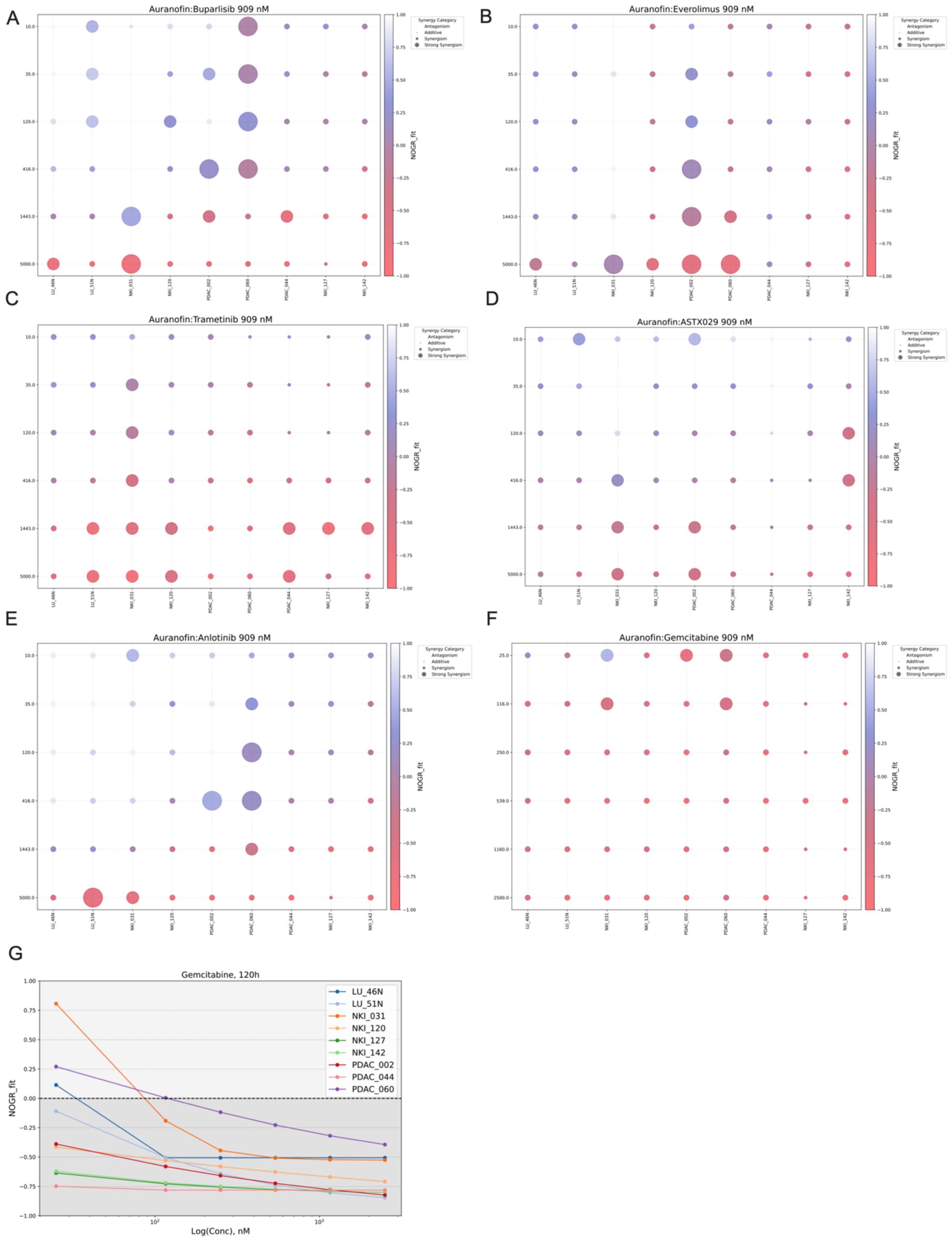

Supplemental Figure 6: Additional Auranofin drug combination strategies. Bubble plots showing the ZIP synergy score (bubble size) and Normalized Organoid Growth Rate (NOGR, colored heatmap) for a concentration range of **(A)** Buparlisib, **(B)** Everolimus, **(C)** Trametinib, **(D)** ASTX029, **(E)** Anlotinib and **(F)** Gemcitabine in combination with 909 nM Auranofin for each organoid line. Bubble size: very small = ZIP <-10 indicating antagonism; small = -10 < ZIP < 10 indicating an additive effect; medium = 10 < ZIP < 20 indicating moderate synergism; large = ZIP > 20 indicating strong synergism. NOGR between 1 and 0 indicates a cytostatic response,

NOGR < 0 indicates a cytotoxic response. **(G)** Fitted dose response curves of the NOGR values for the treatment with Gemcitabine.

Organoid Line	Type	Sampling	Location	Stage	Passage*	Source	Ref
LU_46N	Pulmonary	Resection	Lung	/	4	UA	7
LU_51N	Pulmonary	Resection	Lung	/	6	UA	7
NKI_031	NSCLC	Resection	Lung	II	19	NKI	9
NKI_120	NSCLC	Biopsy	Lymph node	IV	13	NKI	9
NKI_127	NSCLC	Biopsy	Lymph node	IV	18	NKI	9
NKI_142	NSCLC	Resection	Liver	IV	15	NKI	9
PDAC_002	PDAC	Resection	Pancreas	III	7	UA	10
PDAC_044	PDAC	Resection	Pancreas	III	6	UA	10
PDAC_060	PDAC	Resection	Pancreas	III	7	UA	10
PDAC_087	PDAC	Resection	Pancreas	III	8	UA	10

Table S1: Tumor organoid characteristic. * Passage at which organoids were thawed. All organoids were passaged an additional 3-4 cycles before drug screening. (UA: University of Antwerp, NKI: Netherlands Cancer Institute, NSCLC: non-small cell lung cancer; PDAC: pancreatic ductal adenocarcinoma).
